# Modified bilateral fasciaperichondrial flap for prominent ear correction

**DOI:** 10.1016/j.bjorl.2022.01.008

**Published:** 2022-02-15

**Authors:** Abdulhalim Aysel, Berrak Karatan, Uğurtan Ergün, Togay Müderris

**Affiliations:** a Health Sciences University Bozyaka Training and Research Hospital, Department of Otorhinolaryngology and Head and Neck Surgery, İzmir, Turkey; b Izmir Bakırçay University Çiğli Training and Research Hospital, Department of Plastic, Reconstructive and Aesthetic Surgery, İzmir, Turkey; c Izmir Bakırçay University Çiğli Training and Research Hospital, Department of Otorhinolaryngology and Head and Neck Surgery, İzmir, Turkey

**Keywords:** Otoplasty, Prominent ear, Surgical techniques

## Abstract

**Objective:**

Various techniques have been described in the literature for prominent ear correction. These cartilage-preserving or cartilage-shaping techniques have their own advantages and disadvantages. We aim to achieve aesthetic and stable results with low complication rates using combinations of these methods. Herein, we present our results of prominent ear surgery with a modified bilateral fasciaperichondrial flap in combination with concha-mastoid and concha-scaphal sutures.

**Methods:**

Patients whose surgeries included a modified bilateral fasciaperichondrial flap for prominent ear deformities were included in the study. Patients’ demographic data, pre- and postoperative Concha-Mastoid Angle (CMA) and upper-middle Helix-Mastoid Distances (HMD), follow-up time, complications, secondary operations, and postoperative Visual Analogue Scale (VAS) results were evaluated. With a postauricular fish-mouth incision, the bilateral fasciaperichondrial flap was planned into two: proximal- and distal-based. They were then elevated from the cartilage subperichondrially on the proximal side and supraperichondrially on the distal side. Concha-scaphal sutures were used to form an antihelical rim along with concha-mastoid sutures to reduce the concha-mastoid angle. Conchal cartilage resection was done if needed. Then, the bilateral fasciaperichondrial flaps were sutured together to cover the concha-mastoid and concha-scaphal sutures.

**Results:**

Between May 2017 and May 2021, 32 ears of 17 patients were operated on due to prominent ear deformity. No hematoma or infection was observed in any patient, and there were no instances of recurrence, suture exposure, hypertrophic scars, or keloids. The satisfaction level of all patients was 8.2 ± 0.9 points on average according to the VAS. In the anthropometric measurements, a statistically significant difference was found between preoperative and postoperative sixth month CMA and HMD values.

**Conclusion:**

A combination of suture techniques and a modified bilateral fasciaperichondrial flap may be used in prominent ear cases, with low recurrence rates and high patient satisfaction.

**Level of evidence:**

III.

## Highlights

• Prominent ear deformity is the most common congenital head and neck deformity.
• Various techniques have been described for prominent ear correction.
• Modified bilateral fasciaperichondrial flap technique is used with low complications.

## Introduction

The auricle is a flexible organ consisting of concha, helix, antihelix, tragus, and lobule structures formed by skin, muscle, cartilage, and adipose tissues.^1^ Prominent ear deformity is the most common congenital head and neck deformity.^2^ Prominent ear deformity occurs due to overdevelopment of the concha, underdevelopment of the antihelix, or both.^2^ Its incidence in Caucasian populations is 5%, regardless of age and gender, and it is inherited in an autosomal dominant manner.^2^

Prominent ear deformity can cause significant psychological problems in school-age children and adolescents,^3^ and prominent ear surgery is an operation that is frequently performed during childhood.^3^ Because the ear cartilage hardens with age, it may be more difficult to shape and maintain the given shape in adults compared to children.^3^

Although various prominent ear surgery techniques are described in the literature, these techniques can be grouped under two main methods: cartilage-preserving and cartilage-shaping.^4^, ^5^ The literature states that cartilage-shaping methods may result in higher rates of recurrence, hematoma, skin necrosis, sharp cartilage edges, and unnatural appearance compared to cartilage preserving methods.^6^, ^7^ Complications such as exposure of sutures from the skin, granuloma, and keloid formation may occur in cartilage-preserving methods. Today, cartilage-preserving methods are mostly preferred.^6^, ^7^

In cartilage-preserving methods, shaping is done with sutures and avoids incisions and cartilage removal.^4^, ^5^ The best known methods include the Furnas technique, which reduces the concha-mastoid angle and Mustardé technique, which reduces the scapha-conchal angle and is used to create an antihelix.^4^, ^5^ In cartilage shaping techniques, cartilage is shaped by cutting, scratching, or removing a piece of cartilage.^4^, ^5^

While cartilage-preserving methods are thought to cause more recurrence than cartilage-shaping methods, the latter can cause bleeding, hematoma, and other related complications more frequently due to extensive dissection and irreversible changes in the cartilage structure.^4^, ^5^

In prominent ear surgeries, there are interventions made in the soft tissue as well as the cartilage. The most common of these interventions is the postauricular fascial flap, which was first described by Horlock et al. In this technique, the fascial flap is used together with cartilage-preserving and shaping methods.^8^ The flap covers the stitches, thus preventing the stitches from being exposed and avoiding recurrence.^8^ Permanent stitches are not used in some modified flap methods, and the Furnas and Mustardé suture techniques may not always be used.^9^, ^10^

Each method has its own advantages and disadvantages.^8^, ^9^, ^10^ It is still controversial which method will be suitable for each individual patient in prominent ear surgery.^8^, ^9^, ^10^ Many surgeons try to create their own standard by using the method they feel is safest or by using combinations of various methods.

In this study, we present the results of patients who underwent prominent ear deformity repair with a modified bilateral fasciaperichondrial flap.

## Methods

The data of the patients who applied to the Plastic Surgery and Ear-Nose-Throat Clinic of our hospital between May 2017 and May 2021 due to prominent ear deformity were evaluated retrospectively after obtaining approval from our hospital’s Clinical Research Ethics Committee (Decision number: 2021/107). Detailed information about the technique to be performed was given to all of our patients and their parents before surgery, and their consents were obtained. In each case, the details of the ears were observed topographically on both sides, and preoperative-postoperative Concha-Mastoid Angle (CMA) and upper-middle Helix-Mastoid Distance (HMD) were measured. The operation plan was made by evaluating the conchal depth and lobule prominence. Preoperative and postoperative first week, first-month, third month, and six-month photographs of the patients were taken.

The patients’ demographic data, pre- and postoperative HMD (two points: upper and middle) and CMA measurements, follow-up time, early and late complications, need for a secondary operation, and the results of the surgery were evaluated. At the six-month control, patients were asked to evaluate the results of their prominent ear surgery using the Visual Analogue Scale (VAS). On this scale, 1–3 points were evaluated as “very bad”, 4–6 points as “acceptable”, and 7–10 points as “good” or “very good”. Points in the “good” and “very good” ranges were accepted as satisfactory in this study.

Patients with collagen, vascular, and connective tissue diseases; other ear anomalies, including Stahl’s ear, constricted ear, microtia, macrotia, etc.; and revision cases were not included in the study.

### Surgical technique

Pediatric patients were operated on under general anesthesia, and adult patients were operated on under local anesthesia with sedation (Video S1).

Measurement: The HMD was measured from two points: the upper edge of the helix and the projection of the helix to the outermost edge of the concha.

**Figure 1:**
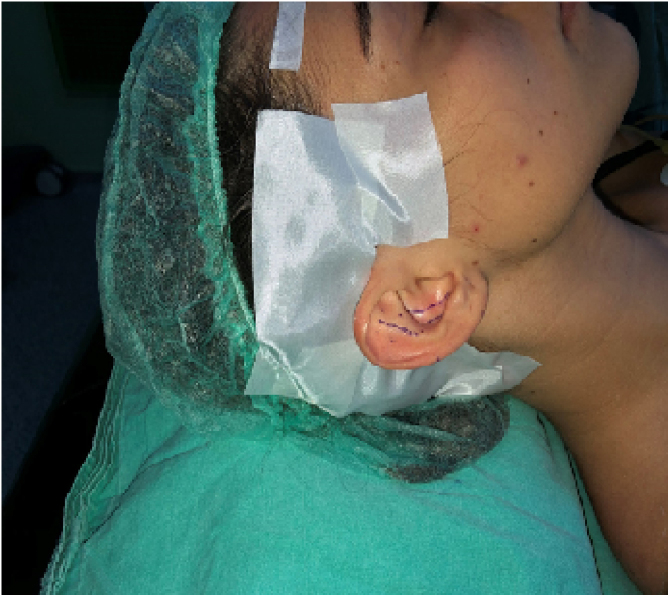
Skin marking.

Marking: The planned antihelix, conchal cartilage to be removed, and postauricular fish-mouth incision were marked (Fig. 1).

Local anesthesia: The auricle was anesthetized with an average of 10 mg of lidocaine and a local anesthetic containing 1/1000 adrenaline.

Incision: A fish-mouth incision was made so as not to create tension in the skin closure, projecting onto the planned antihelix and concha.

Thin skin removal: Very thin skin was removed with a fish-mouth incision, leaving the subcutaneous flap the thickest.

**Figure 2:**
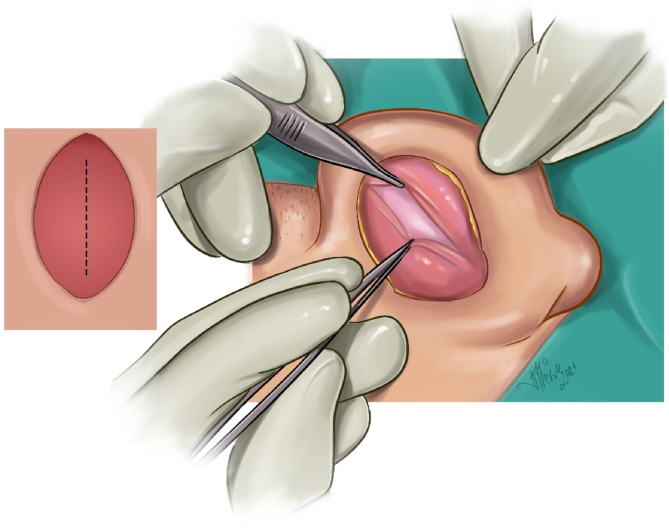
Distal and proximal based fasciaperichondrial flap illusturation.

Creation of bilateral-based fasciaperichondrial flap: The flap consisting of subcutaneous fat, fascia, muscle, and perichondrium was divided into two: proximal- and distal-based. It was then elevated from the cartilage. Proximally, the flap was elevated up to the postauricular sulcus in the subperichondrial plane. Distally, the flap was elevated until the planned antihelix was passed by 5 mm in the supraperichondrial plane, leaving a very thin perichondrium layer on the cartilage (Fig. 2). The posterior auricular muscle was preserved.

**Figure 3:**
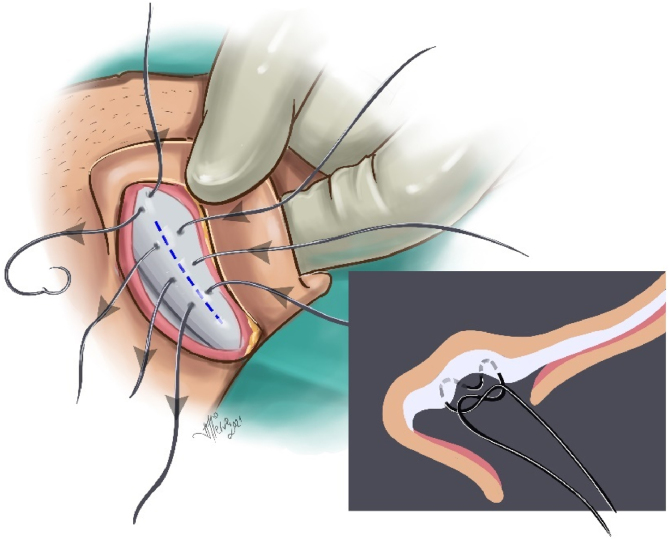
Antihelix creation, Mustardé (horizontal mattress) sutures illusturation.

Creating an antihelix: The planned antihelix was determined with a dental needle tip. In patients with thick cartilage, the antihelix projection was shaved from the posterior approach. Antihelical sutures were created with two or three Mustardé (horizontal mattress) sutures by round needle 3/0 polypropylene (white, colorless). In patients with thinner cartilage, 4/0 polypropylene was used. Care was taken for the sutures to pass through the cartilage and perichondrium on the anterior surface but not through the skin (Fig. 3).

**Figure 4:**
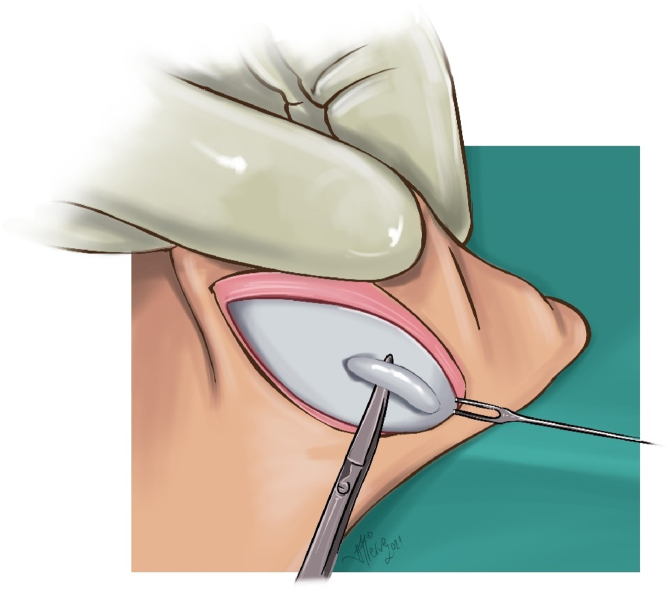
Conchal cartilage excision illusturation.

**Figure 5:**
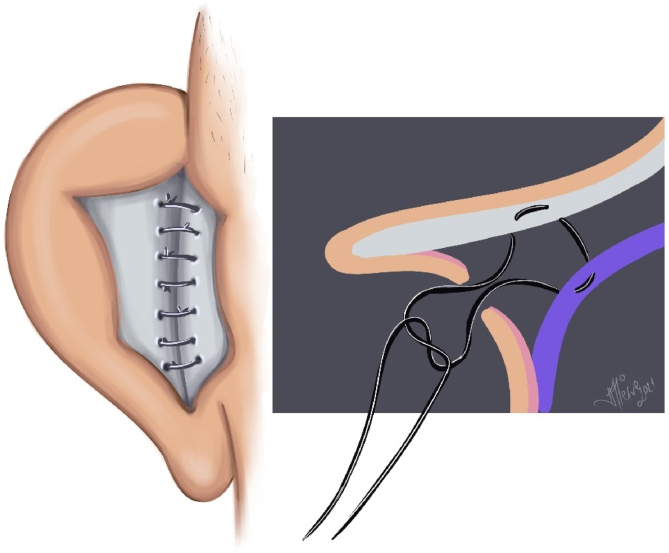
Concha-mastoid (Furnas) sutures demonstration illustration.

Cartilage excision from the auricular concha and reduction of concha-mastoid distance: In all cases with a conchal depth of more than 15 mm, varying amounts of conchal cartilage (average 15 × 5 mm) were excised from the lateral part of the concha (Fig. 4). The medial cartilage was dissected in the subperichondrial plane, and the skin was lifted toward the entrance of the external auditory canal. The excess skin was medialized, and the skin potency was decreased. Then, the cartilage edges were brought closer using 5/0 PDS suture material. The concha-mastoid angle was narrowed by reducing the depth of the concha with two or three concha-mastoid (Furnas) sutures using round needle 3/0 polypropylene (white, colorless) for thick cartilage and 4/0 polypropylene for thinner cartilage and by suturing the proximal flap to the mastoid periosteum (Fig. 5).

**Figure 6:**
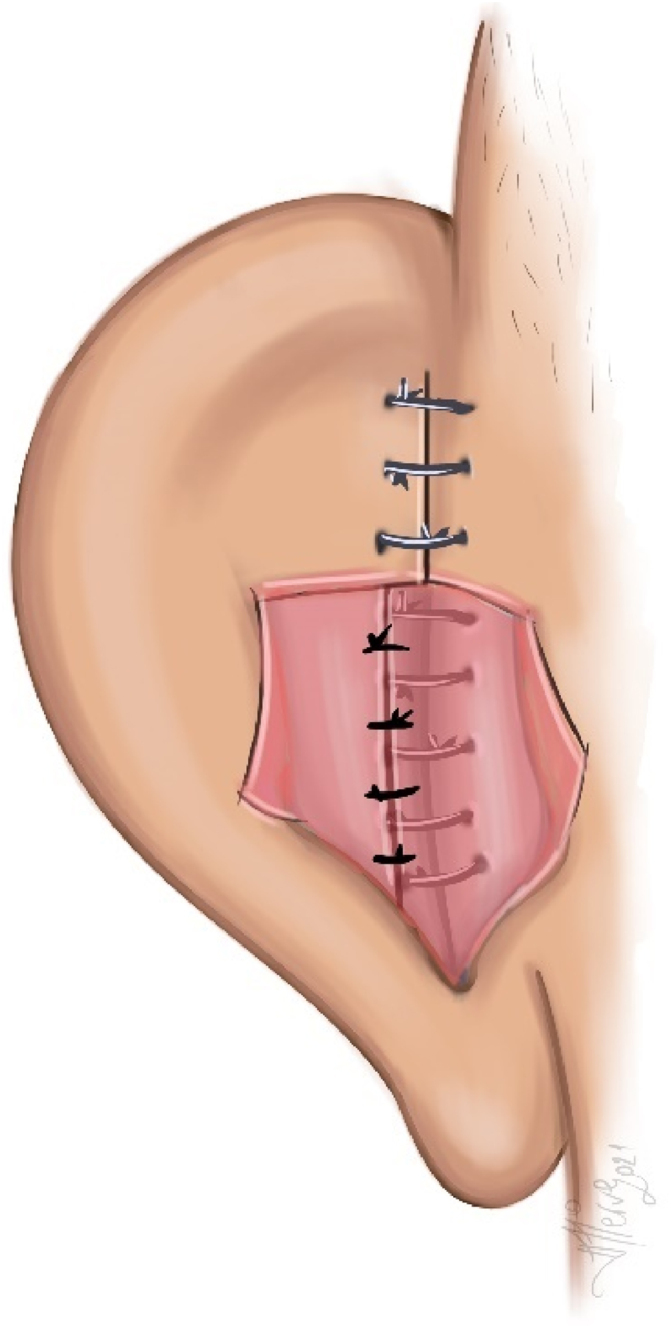
Flap suturation illustration.

Suturing of distal-proximal base flaps: The flaps were sutured with 5/0 rapid polyglactin sutures to cover the Furnas and Mustardé sutures (Fig. 6).

Placing a Penrose drain: A Penrose drain was placed between the flaps inferiorly to prevent hematoma and reduce edema.

Skin suturing: The skin was primarily sutured with 5/0 rapid polyglactin sutures.

Dressing: Mupirocin 2% cream-impregnated tampons were placed on the anterior aspect of the auricle to support the newly formed antihelix and postauricular sulcus and to prevent possible hematoma.

Postoperative care: In all cases, dressings were opened on the first postoperative day, and the wound was evaluated in terms of hematoma, necrosis, and edema. The drain was removed on the second postoperative day. A pressure dressing was used every other day between the third and seventh postoperative days, and the skin sutures were removed on the seventh day. Empirical antibiotherapy and analgesia were used on all of our patients for a week. After one week, it was recommended that patients leave their ears uncovered during the day and use tennis bands for three months while sleeping at night. Patients were routinely invited for controls during the postoperative first, third, sixth month, first year, and second year.

### Statistical analysis

Statistical analysis was done using IBM SPSS Statistics 21. An independent sample *t*-test was applied for variables consisting of independent groups according to the results of the Shapiro-Wilk normality test; a paired sample *t*-test was applied to variables consisting of dependent groups. Results of p < 0.05 were considered statistically significant.

## Results

Between May 2017 and May 2021, 32 ears of 17 patients were operated on due to prominent ear deformity. The ages of the patients included in the study were between 6 and 27 years (mean ± SD; 17.2 ± 5.9 years). Nine (52.9%) of the patients were female and 8 (47.1%) were male. Bilateral prominent ear correction was performed on 15 (88.2%) patients, and unilateral prominent ear correction was performed on 2 (11.8%) patients. While the primary problem in all cases was insufficient development of the antihelix, the flat and deep auricular concha was an additional problem in 14 (82.3%) patients.

While the conchal cartilage was not removed in three of the patients, approximately 15 × 5 mm of cartilage was removed from the conchal cartilage lateral margin in fourteen patients. Mustardé and Furnas techniques were used in all patients.

Postoperative follow-up time ranged from 6 months to 46 months, with a mean of 23.6 ± 13.1 months. The demographic and clinical characteristics of the patients are presented in Table 1.

**Table 1:** Demographics and clinicals features of patients.

Age (min‒max, mean ± SD)	6‒27; 17.2 ± 5.9
Gender ‒ F, n (%)/M, n (%)	9 (52.9%)/8 (47.1%)
Side ‒ Right, n (%)/Left, n (%)/Bilateral, n (%)	1 (5.9%)/1 (5.9%)/15 (88.2%)
VAS (min‒max/mean ± SD)	7‒9/8.2 ± 0.9
Follow-up (min‒max/mean ± SD)	6‒46, 23/6 ± 13.1

F, female; M, male; VAS, visual analog scale.

No recurrence of prominent ear deformity was observed in any patient during the follow-up period. There was no hematoma or infection in the early postoperative period. Suture exposure was not observed in any patient. Hypertrophic scars, keloids or skin necrosis did not develop in any patient during the postoperative follow-up period.

**Figure 7:**
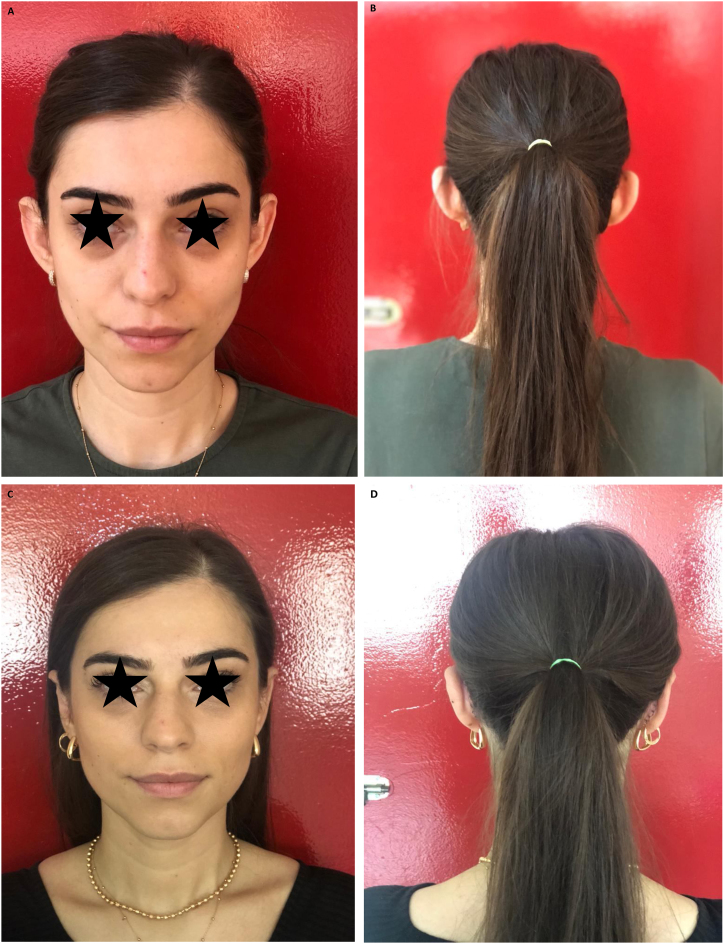
Bilateral prominent ear correction; A 23-year-old female patient. (A), Preoperative anterior view. (B), Preoperatif posterior view. (C), Postoperative anterior view. (D), Postoperative posterior view.

The satisfaction level of all patients was 8.2 ± 0.9 points on average according to the VAS. All patients were satisfied with the results (Fig. 7).

The average preoperative measurements of the upper-middle HMD for the right ear were 30.8 ± 5.2 mm and 28 ± 5.1 mm, while the average six-month postoperative measurements were 16.5 ± 1.7 mm and 14.1 ± 1.6 mm (*p* <  0.05, *p* <  0.05, respectively). For the left ear, the average preoperative measurements of the upper-middle HMD were 29.9 ± 4.8 mm and 27 ± 4.8 mm, while the average six-month postoperative measurements were 15.6 ± 1.9 mm and 13.7 ± 1.9 (*p* <  0.05, *p* <  0.05, respectively). The mean preoperative measurements of the CMA were 45.9 ± 8.7 for the right ear and 44.5 ± 8.6 for the left ear. For the six-month postoperative measurements, the mean CMA was 20.7 ± 2.9 for the right ear and 19.6 ± 2.7 for the left ear. In the anthropometric measurements, a statistically significant difference was found between the preoperative and sixth-month postoperative HMD and CMA measurements.

## Discussion

Anatomical defects in the prominent ear may include indistinctness in the antihelical fold, an overdeveloped or deep concha, enlargement in the auriculo-cephalic angle, and lobule abnormalities.^2^ The main goals in prominent ear correction are to reduce the helix-mastoid distance and concha-mastoid angle so that the two ears are symmetrical.^2^ The standard for this distance and angle is generally considered to be between 1.5‒2 cm and less than 30°,^2^ which we were able to achieve in all ears in our patient group.

Many techniques have been described in the literature for prominent ear correction. One technique may not be applicable to all prominent ear patients, and sometimes more than one technique can be applied in a single case. What is important is that the surgical procedure results in minimal complications and a good result that satisfies the patient.^2^

The most frequently used suture techniques in cartilage-preserving methods are the Mustardé (concha-scaphal) and Furnas (concha-mastoid) techniques.^11^, ^12^ We used the Mustardé suture technique to create the antihelical fold among our patients. Additionally, in patients with thick and strong cartilage, we eroded the posterior of the antihelical projection. In the Furnas suture technique, the conchal cartilage is sutured to the fascia over the mastoid using non-absorbable sutures. For patients with excessive and deep conchal folds, sometimes full-thickness cartilage removal is performed or a cartilage incision is made from the conchal cartilage to reduce the fold.^11^

We first created a proximal-based flap in the subperichondrial plane to reduce the concha-mastoid angle and distance, thus protecting the posterior auricular muscle. In cases with more conchal cartilage folds and depth, we excised the lateral portion of the cartilage in the form of a crescent. We then separated the skin from the cartilage toward the medial side to prevent skin slackening and approached the concha cartilage edges with a 5/0 polydioxanone suture. Then, we sutured the concha to the mastoid periosteum together with the proximal flap with 2–3 non-absorbable 3/0 or 4/0 colorless polypropylene sutures.

The use of the postauricular fascial flap contributes to the success of the suture methods used in antihelix formation. This, in turn, helps reduce recurrence and complications.^8^, ^13^, ^14^ In addition to contributing to the retraction of the ear, this flap also prevents the sutures from being exposed by covering the permanent stitches.^8^, ^13^, ^14^ In our technique, we covered the Mustardé sutures with a distal-based flap and the Furnas sutures with a proximal-based flap. We also protected the posterior auricular muscle. Studies conducted on large patient groups in which postauricular fascial flap techniques are applied also support the decrease in recurrence and complication rates.^8^, ^13^, ^14^ We did not observe any sutures exposed from the skin, keloid formation, or recurrence in any of our patients. We believe that, with the use of this flap that we defined, complications such as exposure of the sutures or appearance of the suture from the skin due to the permanent suture materials used in Mustarde and Furnas suture techniques are reduced.

Various modified facial flap techniques have been described in the literature. The primary purpose of these techniques is to reduce concha-mastoid, scapha-conchal, and scapha-mastoid angles. Flap elevation can be done sub or supraperichondrially.^8^, ^13^, ^14^, ^15^, ^16^ For instance, Basat et al. reported in their study that they reduced the risk of suture exposure from the skin by covering the Mustardé and Furnas sutures with a distal-based fascial flap elevated in the supraperichondrial plane.^15^

In their study, Ersen et al. created a U-shaped, distal-based subperichondrial flap and dissected the proximal and mastoid region in the supraperichondrial plane. This created a subdermal nest in the postauricular sulcus, and by using 4-0 poliglecaprone sutures from three points, they reduced the concha-mastoid angle and distance. They did not use the Furnas and Mustardé techniques.^16^ They reported that they did not experience early-term complications such as hematoma, skin necrosis, and suture removal from the skin, and their long-term revision rate was 12.3% (20/162).^16^ Cihandide et al. also created a distal-based facial flap in the subperichondrial plane. They then sutured the flap to the mastoid fascia with 4.0 polypropylene sutures at two points, thereby reducing the concha-mastoid angle and distance. They also did not use the Furnas and Mustardé techniques.^17^ In the long-term follow-up, a revision was planned in one patient (1/20, 5%) due to asymmetry, but the patient did not want it.

Irkoren et al. applied the Mustardé and Furnas suture techniques in order to create an antihelix and reduce concha-mastoid distance and angle after bilateral (distal-proximal) fasciaperichondrial flap elevation in the subperichondrial plane. In addition, in the thick, resistant cartilage, the resistance of the cartilage was reduced by crossing the cartilage with the help of a needle tip and a small incision in the anterior in the projection of the antihelix.^18^ After the suture techniques were applied, the double flap was mutually sutured.^18^ Our technique differed in that the distal flap was elevated in the supraperichondrial plane because, when it is lifted in the subperichondrial plane and the Mustardé suture technique is applied, the cartilage may break down and become very difficult to repair. As a matter of fact, while in the subperichondrial plane in one of our patients, the cartilage was damaged while performing the Mustardé technique, so one missing Mustardé suture was applied. As a solution, the distal flap part corresponding to the cut cartilage was divided into two and sutured more proximally. In addition, matrix fixation sutures passing externally through the skin were placed.

In their technique using a proximal-based fascial flap in the supraperichondrial plane to create an antihelical rim, Tas et al. did not use the Mustardé suture technique or permanent sutures. Instead, they used the Furnas suture technique with 3.0 absorbable polydioxanone suture material and excised the cartilage in cases with large concha depth and curvature.^19^ They did not experience complications in the early or late periods of their study, which included 24 patients.^19^ In some studies comparing the suture materials used in prominent ear deformity repair, the revision rates were higher in cases where absorbable suture materials were used.^20^ We used 3.0 and 4.0 colorless non-absorbable polypropylene along with the Mustardé and Furnas suture techniques.

Brenda et al. recommend some over-correction after Mustardé sutures are placed due to “cartilage memory” in the cartilage,^21^ as we did in all of our cases. They stated that the ears were lateralized somewhat in the recovery period over time and that there was an increase in HMD and CMA measurements.^21^

The small number of patients and a relatively short follow-up period are the limitations of our study.

## Conclusion

We used more than one surgical technique with each patient along with a modified bilateral fasciaperichondrial flap. The absence of recurrence and high patient satisfaction rates suggests that a combination of suture techniques and a modified bilateral fasciaperichondrial flap may be used in prominent ear cases, with low recurrence rates and high patient satisfaction.

## Conflicts of interest

The authors declare no conflicts of interest.

## Appendix A Supplementary data

Video S1 Right prominent ear correction with modified bilateral fasciaperichondreal flap.
